# Large scale study of anti-sense regulation by differential network analysis

**DOI:** 10.1186/s12918-018-0613-7

**Published:** 2018-11-20

**Authors:** Marc Legeay, Sébastien Aubourg, Jean-Pierre Renou, Béatrice Duval

**Affiliations:** 1 LERIA, Université d’Angers, 2 bd Lavoisier, Angers, 49045 France; 2 IRHS, Agrocampus-Ouest, INRA, Université d’Angers, SFR 4207 QuaSaV, Beaucouzé, 49071 France

**Keywords:** Differential network analysis, Anti-sense regulation, Functional analysis

## Abstract

**Background:**

Systems biology aims to analyse regulation mechanisms into the cell. By mapping interactions observed in different situations, differential network analysis has shown its power to reveal specific cellular responses or specific dysfunctional regulations. In this work, we propose to explore on a large scale the role of natural anti-sense transcription on gene regulation mechanisms, and we focus our study on apple (*Malus domestica*) in the context of fruit ripening in cold storage.

**Results:**

We present a differential functional analysis of the sense and anti-sense transcriptomic data that reveals functional terms linked to the ripening process. To develop our differential network analysis, we introduce our inference method of an Extended Core Network; this method is inspired by C3NET, but extends the notion of significant interactions. By comparing two extended core networks, one inferred with sense data and the other one inferred with sense and anti-sense data, our differential analysis is first performed on a local view and reveals *AS-impacted genes*, genes that have important interactions impacted by anti-sense transcription. The motifs surrounding AS-impacted genes gather transcripts with functions mostly consistent with the biological context of the data used and the method allows us to identify new actors involved in ripening and cold acclimation pathways and to decipher their interactions. Then from a more global view, we compute minimal sub-networks that connect the AS-impacted genes using Steiner trees. Those Steiner trees allow us to study the rewiring of the AS-impacted genes in the network with anti-sense actors.

**Conclusion:**

Anti-sense transcription is usually ignored in transcriptomic studies. The large-scale differential analysis of apple data that we propose reveals that anti-sense regulation may have an important impact in several cellular stress response mechanisms. Our data mining process enables to highlight specific interactions that deserve further experimental investigations.

**Electronic supplementary material:**

The online version of this article (10.1186/s12918-018-0613-7) contains supplementary material, which is available to authorized users.

## Background

Understanding cell regulation mechanism is a key issue in bioinformatics. In the last two decades, large-scale gene expression profiling has led to considerable advances on this topics. Many discoveries have been obtained by differential expression analyses. In medicine, molecular classification of diseases may be achieved by looking for differentially expressed genes when comparing microarray datasets from healthy and disease samples [1]. Even if useful as prognostic tools, these approaches do not provide explanations about the dysfunctional mechanisms that cause the disease. Therefore, as reviewed in [2], recent works move to differential networking in order to identify cell regulations that are altered in disease samples.

Differential network biology [3, 4] refers to a set of works that rely on differential network mapping to analyse interactions between components of a biological system. These works study the changes that can be observed in the interaction networks representing biological systems when different environmental conditions, different tissue types, different disease states or different species are considered.

One type of approach in network biology is to integrate static interaction knowledge with dynamic changes in gene expression or metabolic fluxes. In [5], the authors propose to screen a known molecular interaction network to identify active sub-networks; an active sub-network is a connected region of the network with an unexpected high level of differential expression over particular sets of conditions. The top-scoring active sub-networks help to uncover regulatory mechanisms that control the expression changes in these experiments. In [6], the integration of a static protein-protein interaction network with ageing-related gene expression data is used to identify cellular changes related to age. An interaction from the reference network is put in the network associated to age *A* if the two related proteins are expressed in the datasets relative to *A*. The topologies of 37 different age-specific networks are analysed. While the global network topology does not exhibit significant changes with age, the measures of local topology (node degree, clustering coefficient, graphlet degree, …) reveal a small set of proteins whose centrality values are correlated with age.

Another possible approach is to compare networks inferred from data. A lot of methods have been proposed in the literature for the task of building an interaction network from a collection of expression data [7–10]. In these reverse engineering methods, the network modelises gene interactions as static processes inferred from a set of similar samples. To decipher the cellular response to different situations, more recent studies propose to identify differential co-expression patterns by comparing several networks [11, 12]. The comparison may be carried on pair-wise gene co-expressions by quantifying the significant differences [13, 14]. The comparison may also identify sub-networks containing significant changes of regulation; in [15], the authors measure the preservation of network modules across a set of condition-specific networks. Thus differential network analysis proposes to identify significant interaction modifications when comparing networks involving the same set of actors but related to different conditions.

The present work aims to study the impact of anti-sense transcription on gene networks by a method inspired by differential network analysis. Anti-sense RNAs are endogenous RNA molecules whose partial or entire sequences exhibit complementarity to other transcripts. Their different functions are not completely known but several studies suggest that they play an important role in the regulation of gene expression [16]. Mechanisms of anti-sense regulation can affect positively or negatively the protein production through their impact on transcription [17], mRNA degradation/stability [18] or final translation [19]. More recent works have also shown that anti-sense RNA are involved in the chromatin architecture [20]. Among their different functions, anti-sense genes can trigger the post-transcriptional gene silencing: it is a RNA-degradation mechanism creating small interfering RNAs (siRNA). The anti-sense transcript and the sense transcript hybridize themselves to form a double strand RNA (dsRNA). For instance, in *Arabidopsis thaliana*, [21] found that the RPP5 defence gene was affected by this phenomena: its sense and anti-sense dsRNA are degraded in siRNA which presumably contributes to the degradation of the sense transcript in the absence of pathogen infection. A similar mechanism regarding the salt tolerance in *Arabidopsis thaliana* has been described [22]. The description of these regulations and the results of genome-wide approaches [23, 24] suggest that anti-sense genes and RNA are major actors of biological pathways and must be integrated in the methods inferring gene networks.

The present work deals with apple data because a previous study [24] reveals different interesting points about anti-sense transcription in this organism. The authors used a dedicated microarray chip and RNA-Seq of small RNAs to analyse anti-sense transcription in eight different organs of apple *Malus domestica*. In their analysis, the three following points are important results about anti-sense transcription. Firstly, their measures of sense and anti-sense transcription show that, when considering the sense genes expressed in at least one of the eight organs, a significant level of anti-sense expression is found for 65% of them. This observation is higher than the previous studies performed on *Arabidopsis thaliana* [25, 26] that identified only 30% of anti-sense expression. Secondly, the presence of short interfering RNAs is correlated with the anti-sense transcript expression. Thirdly, the levels of expression of anti-sense transcripts vary depending on both organs and Gene Ontology (GO) categories: genes related to fruits and seeds, and belonging to the "defense" GO class have higher levels.

The work described in this paper proposes a genome-wide analysis of apple transcriptomic data, with measures of anti-sense transcripts, in the context of fruit ripening and cold storage. To identify the impact of integrating anti-sense transcription in gene network inference, we propose to achieve an original differential network analysis where we compare two context-specific gene networks inferred from two sets of actors: the first set is only composed by sense transcripts, it represents a usual transcriptomic study; the second set is composed by both sense and anti-sense transcripts. By computing the major differences between these two networks, we aim to highlight interactions that are greatly impacted by anti-sense transcripts and that would be neglected in a classical gene expression analysis.

**Fig. 1:**
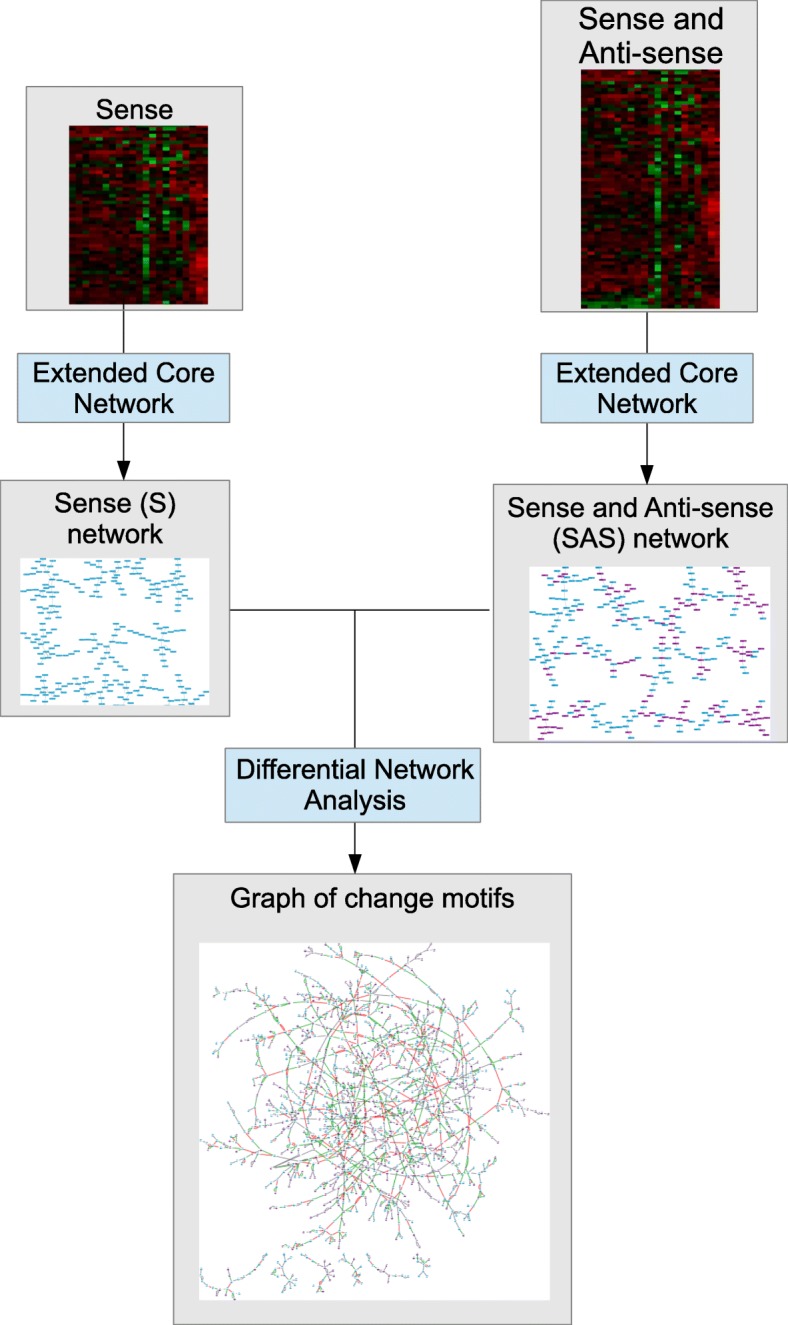
Differential network analysis. In order to identify change motifs, we realise a differential network analysis from gene networks inferred by Extended Core Network on Sense data in one hand, and Sense and Anti-Sense data in an other hand

The gene network inference methods based on statistical measures lead to infer many interactions in the network. Some of them are noise or redundant, we call them false positive interactions. A lot of works have been done to minimize such false positive interactions [27, 28]. Altay and Emmert-Streib [8] proposes to study what they call the core part of the gene network. The core network represents the most trustworthy part of the network: it is computed by selecting only the most significant interaction for each gene. However, the constraint of selecting only one interaction per gene seems too restrictive. We propose to extend the core network by considering a small number of significant interactions for each gene. Therefore, our gene network inference method computes what we call an *Extended Core Network (ECN)*. We use ECN in our differential analysis to discover the interactions of the core network that are impacted by the integration of the anti-sense transcripts. Our proposal is summarized by the workflow presented in Fig. 1. We then define the notion of *AS-impacted genes* to identify the genes whose network neighbourhood is drastically different when the anti-sense transcripts are considered. We also study the relationships between the AS-impacted genes and we try to explain how their neighbourhood is rearranged by computing Steiner trees.

The rest of the paper is organized as follows. In “Methods” section, we first present the biological samples and the differential functional analysis that we propose to study the impact of anti-sense data on functional enrichment tests. Then we introduce the Extended Core Network Inference algorithm and we evaluate it on artificial datasets. We also present our differential analysis of two core gene networks built on different sets of actors, with the associated definition of change motifs and AS-impacted genes. We finally explain the computation of Steiner trees to study the relationships between AS-impacted genes. In “Results and discussion” section, we first present the results of the differential functional analysis ; the outputs are functional terms clearly consistent with ripening in cold situation. We then present the results of our differential network study, with a detailed biological analysis that confirms the interest of taking into account anti-sense transcription in biological experiments.

## Methods

### Biological material

We study the anti-sense transcription in the context of apple fruit ripening thanks to microarray data. The AryANE v1.0 microarray is designed to detected 63,011 predicted sense genes and 63,011 complementary anti-sense sequences for the *Malus domestica* genome [29]. With this microarray, we can identify the sense and anti-sense expression for the whole predicted genome. We used data produced in order to study the evolution of fruit in cold storage condition for different varieties. For each 22 genotypes we have two samples: one at harvest time (H) and one 60 days after harvest time (60DAH) with fruits kept at 4°C. The data produced by the microarray are the intensity values of loci, a locus being associated to a gene. In order to analyse the microarray data, we use the quantile normalization [30, 31]. Then we selected differentially expressed transcripts (*p*-val < 1%) between harvest and 60 days later. Based on a previous study [24], we applied a further threshold of 1 log change between the two conditions. We identified 931 sense transcripts and 694 anti-sense transcripts differentially expressed between H and 60DAH. We found 200 couples (i.e. sense and anti-sense complementary transcripts) among the differentially expressed transcripts. We use all these 1,625 transcripts into our analysis.

### Differential functional analysis

As we observe a significant number of anti-sense transcripts in the differentially expressed probes, we first try to investigate the biological functions concerned by these actors. For that, we perform a differential functional analysis [32] with the particularity to analyse on one hand the sense data, and on the other hand the sense and anti-sense data.

Functional enrichment is used in order to identify biological functions associated to a set of genes. It relies on an ontology that regroups and hierarchizes a set of terms associated to genes. An ontology is an acyclic oriented graph, linking terms with a subsumption relation. Terms are ordered from the most specific ones to the most generic one.

The Gene Ontology Consortium provides three independent Gene Ontologies (GO) : “molecular function”, “biological process” and “cellular component” [33]. In a Gene Ontology, the most generic term is the one named after the ontology. The biochemical activity of the gene’s product is stored in the “molecular function” GO. It only specifies the activity, not where nor when it happens. A “biological process” GO term refers to a process in which the gene is involved. A biological process is an association of several molecular function *via* chemical or physical transformations. The “cellular component” GO indicates where the product is active.

In order to identify biological functions, we will use the “biological process” Gene Ontology and thus we associate each gene with a GO term. As anti-sense transcripts are not annotated, we affect to each anti-sense the annotation of the corresponding sense gene. This decision is based on the fact that due to its sequence complementarity, an anti-sense transcript may interact with the corresponding sense transcript, or at least with a very close member of the gene family. In [29], an apple gene is annotated using predicted orthologs (closest homolog) from *Arabidopsis thaliana* [34], the most studied model plant genome. When an apple gene has no homolog, we associate the term “unknown biological processes” to the gene and its anti-sense.

GO slim gives a broad overview of the ontology content without the detail of the specific fine-grained terms which are not always known. The categories were chosen to provide a broad representation of the distribution of biological roles. An annotation file associating a GO slim and a GO term with each of the 126,022 apple genes has also been created.

Once we can associate genes with GO terms, we can perform the functional analysis in which we identify statistically over-represented GO terms in a set of genes. The test performed is a hypergeometric test. A GO term is considered as statistically over-represented if the *p*-value is below a given threshold, generally set to 0.05.

Many tools were developed to identify the statistically over-represented GO categories in a set of genes. Among them, the Cytoscape App named BiNGO [35] provides a visual representation of the ontology: one possible output of BiNGO is a graph with nodes representing over-represented GO categories and arcs representing the hierarchy between them. The graph also represents the proportion of genes associated to a GO category by the size of a node, and the colour of a node codes for the associated *p*-value of the over-representation. BiNGO uses a Bonferroni correction on the hypergeometric test results. This correction is needed to compare the *p*-values obtained for each GO categories.

Concerning the apple ripening condition that we study, we notice an important proportion of anti-sense transcripts that are differentially expressed: the set *AS* is composed of 694 different transcripts whereas the *S* set is composed of 931 different transcripts. Therefore it is relevant to question the role of these anti-sense actors in the ripening process under cold storage conditions. To explore this question, we propose a differential functional analysis.

We perform the differential functional analysis as follows. We compute the over-represented GO categories on the sense set (*S*), then we compute the over-represented GO categories on the sense and anti-sense set (*SAS*=*S* ∪ *AS*). The use of BiNGO to perform the test gives us two sub-graphs of the GO. We propose to compute the difference of these two sub-graphs. We define the *revealed-by-AS* terms as the functional terms of this difference: they represent the functional terms that are outputted from the ontological analysis only when the *AS* transcripts are included.

### Extended core network inference method

#### Motivations

Gene network inference from transcriptomic data has been studied in several works [7, 9, 10]. Some inference methods propose to reconstruct pairwise gene interaction networks by using a statistical measure to infer the co-expression or co-regulation between two genes. This statistical criterion can be Pearson or Spearman correlation [36], or mutual information [8, 27] to detect non-linear relationships. A threshold of significance is used to keep relevant relations in the network. The inconvenient with these methods is that they predict many false positive interactions. Among these false positive interactions, we find interactions that are not biologically true, and non-direct interactions. An indirect interaction can be observed if, for example, two genes *g*_2_ and *g*_3_ are under the regulation of a third gene *g*_1_; in such a situation, the statistical criterion value between *g*_2_ and *g*_3_ is probably high and thus the method will infer an indirect interaction between *g*_2_ and *g*_3_. With these indirect interactions, the network becomes more complex and difficult to interpret. Therefore some pruning methods have been proposed to eliminate them [27]. In [8], the authors deal with this problem by proposing the Conservative Causal Core Network (C3NET) that computes the core of a gene network. The core of the network contains only the strongest interactions of the gene network: for each gene, it selects the best interaction (i.e. with the maximal mutual information). The aim of our method is to identify significant changes in the interactions when anti-sense actors are integrated by comparing two inferred networks. Therefore, we can work with the core of a gene network. However, the C3NET definition of a core network may be too restrictive since several mutual information values may be close to the maximal. This is why we propose a gene network inference method named *Extended Core Network (ECN)* that considers for each gene the most significant interactions.

#### Algorithm

Extended Core Network estimates the gene connections using mutual information. We first copula-transform [37, 38] the data to have a better estimation of the mutual information. The mutual information *M*[*i*,*j*] between genes *i* and *j* is estimated with the same estimator used in C3NET:

$$M[\!i,j] = \frac{1}{2}\log\left(\frac{\sigma_{I}^{2} \sigma_{J}^{2}}{|C|}\right)$$

where $\sigma _{I}^{2}$ and $\sigma _{J}^{2}$ are the variance of the expression vectors *I* and *J* of genes *i* and *j* respectively, and |*C*| is the determinant of the covariance matrix.

The statistical significance of pairwise mutual information is test by re-sampling methods, as C3NET or ARACNE [27]. Values that are not significant are set to 0 before applying the inference algorithm.

The Extended Core Network (ECN) algorithm infers a gene network represented by an adjacency matrix. Given a set of genes *G*, the first step of the algorithm is to create the zero matrix representing the fact that no genes are connected. The second step aims to identify the neighbourhood of each gene, composed of all the genes for which the mutual information value is maximal. In order to compute the maximal values of the mutual information, we use an accepting rate *r*. Given a gene *g*∈*G*, *g*^′^∈*G* is a neighbour of *g* if the mutual information between *g* and *g*^′^ is close to the best mutual information value of *g* with respect to the accepting rate *r*. The accepting rate must be between 0 and 1 where 0 means that we accept nothing but the best neighbour and 1 means that we accept every neighbour with a significant interaction. With an accepting rate fixed at 0, ECN works approximately as C3NET does, the difference is that C3NET selects only one interaction whereas ECN accepts all the interactions in case of two identical best values. An accepting rate fixed at 1 means that we define the core network by selecting all the significant mutual information values of the matrix. This is why the preprocessing step of the mutual information matrix sets all non-significant values to 0.

The mutual information between *g*_1_ and *g*_2_ is the same as the mutual information between *g*_2_ and *g*_1_. So C3NET outputs a symmetrical adjacency matrix, leading in an undirected graph. However we want to compare two core networks and be able to identify the interactions that changed when the anti-sense transcripts are added into the data. This is why our algorithm outputs a asymmetrical adjacency matrix, allowing us to identify the significant modifications in the neighbourhood of a gene.

The complexity of the ECN algorithm is $\mathcal {O}(n^{2})$, where *n* is the number of genes; it is the same as C3NET complexity [8].

#### Evaluation on artificial datasets

In order to compare our Extended Core Network algorithm with C3NET, we use simulated data. The assessment of a network inference method requires a reference biological network; for some organisms like *E. coli* [39, 40] and *S. cerevisiae* [41], gene networks of reasonable size are considered as established knowledge and are used as benchmarks in the following way. From the reference network, a sub-network of the desired size is extracted; then artificial expression datasets are produced by simulating the activity of the chosen sub-network; the inference method is applied on these simulated data and produces a learned network that can be compared to the original network.

We generate our simulated data with sub-networks of *E. coli* and *S. cerevisiae* thanks to the SynTREN [42] generator and simulator. The sub-networks are randomly generated using the *neighbor addition* method which creates a connected network of *n*=200 genes. We use SynTREN to create a dataset *X* of *p*=100 samples by simulating the activity of the genes from the selected network.

To evaluate the error rate of each inference method, we compare the inferred edges with the edges in the true gene network. This comparison gives us the $\text {precision} = \frac {\text {true positive}}{\text {true positive} + \text {false positive}}$ and $\text {recall} = \frac {\text {true positive}}{\text {true positive}+\text {false negative}}$ of each method. The precision and recall are used to compute the error measure named *F*_1_ score such that $F_{1} = 2 \cdot \frac {\text {presicion} \cdot \text {recall}}{\text {precision} + \text {recall}}$.

The inference methods are tested in *S*=500 simulations. A simulation *k*∈[ 1..*S*] is run with a specific dataset *X*_*k*_ formed by *n* genes and $j \in \left [\!\frac {p}{2}..p\right ]$ samples randomly selected from the dataset *X* generated by SynTREN.

We compare different accepting rates of Extended Core Network with C3NET on two datasets, one from *E. coli* and another from *S. cerevisiae*. We test ECN with accepting rates ranging from 20 to 100% with a 10% step and from 0 to 20% with a 1% step.

**Fig. 2:**
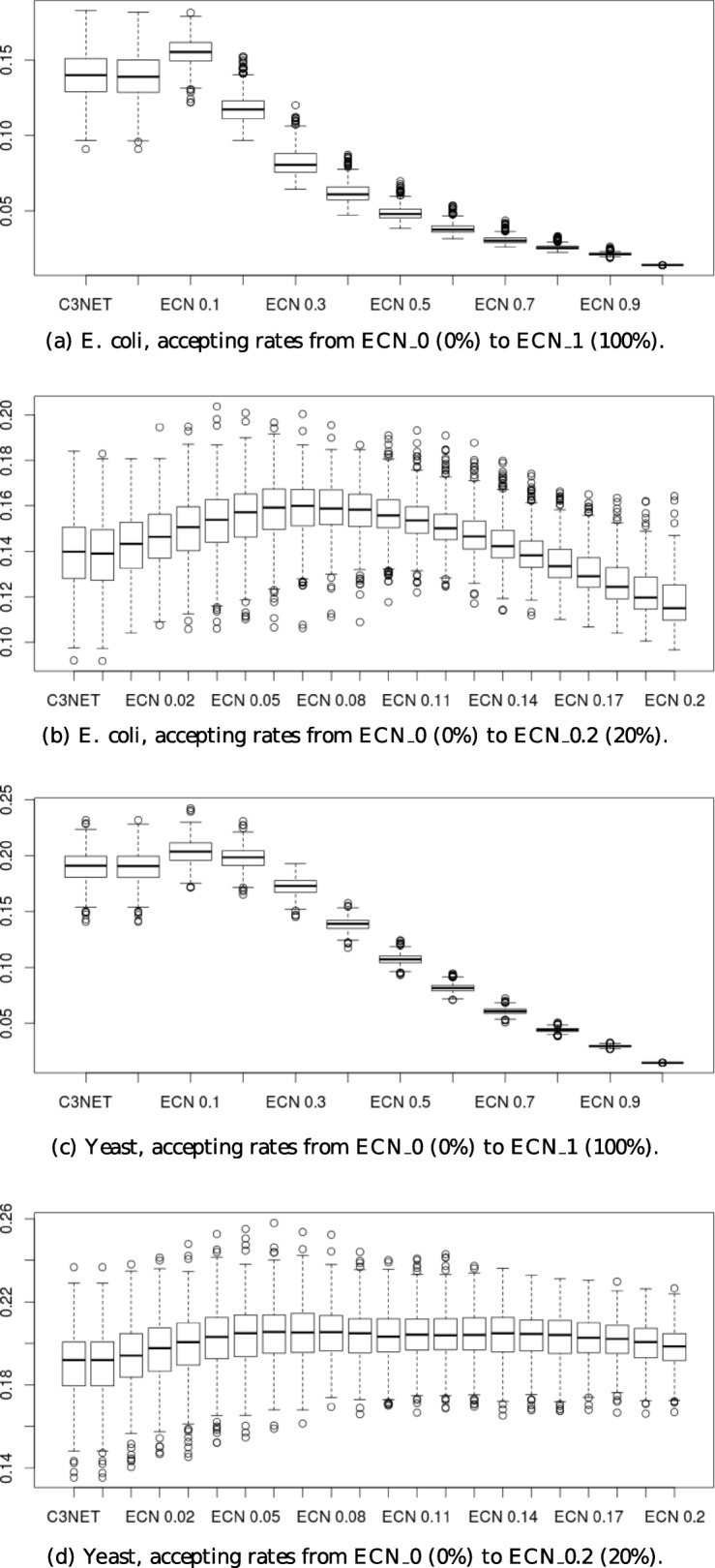
Box plots of *F*_1_ scores for C3NET and ECN with different accepting rates. The number following ECN indicates the accepting rates. The C3NET method is the first on the left, then the ECN methods are sorted beginning with ECN_0. Box plots are obtained from 500 simulations on two datasets : *E. coli* (**a** and **b**) and *S. cerevisiae* (**c** and **d**). Accepting rates from 0 to 100% with a 10% step are tested (**a** and **c**), and from 0 to 20% with a 1% step (**b** and **d**)

Figure 2 shows the box plots of the *F*_1_ scores obtained by the simulations. To enable the comparison with C3NET, ECN is used to produce an undirected network. The ECN_0 method corresponds to the Extended Core Network with a 0% accepting rate. The difference between ECN_0 and C3NET occurs when at least two genes have the best mutual information with one gene. We can observe that ECN has a higher *F*_1_ score than C3NET on *E. coli* (Fig. 2a and b) and yeast (Fig. 2c and d) when the accepting rate is low. As expected, the *F*_1_ score of ECN depends on the values of the accepting rate. For low values, a rate increase improves the score, but when the accepting rate exceeds a certain value, the *F*_1_ score decreases. As explained before, a high accepting rate entails an increased number of false positives, and we observe that it greatly impacts the *F*_1_ score. We can see this phenomena in Fig. 2a and c once the accepting rate is higher than 10% and 20% respectively, the *F*_1_ scores drop down to 0. From empirical observations on several simulated datasets, we can notice that an accepting rate between 5 and 10% is a good rate. In the Fig. 2b and d, we can observe that the rate of 7% (ECN_0.07) is the best rate value on those specific data.

### Differential network analysis

In this section we present the methodology that we propose in order to decipher the impact of anti-sense transcription on gene co-expression network. This methodology relies on the comparison of two extended core networks that reveals *AS-impacted genes* and *change motifs* that we define below. By computing Steiner trees, we also observe how the interactions between AS-impacted genes are reorganized in the network containing sense and anti-sense data.

#### AS-impacted genes and change motifs

**Fig. 3:**
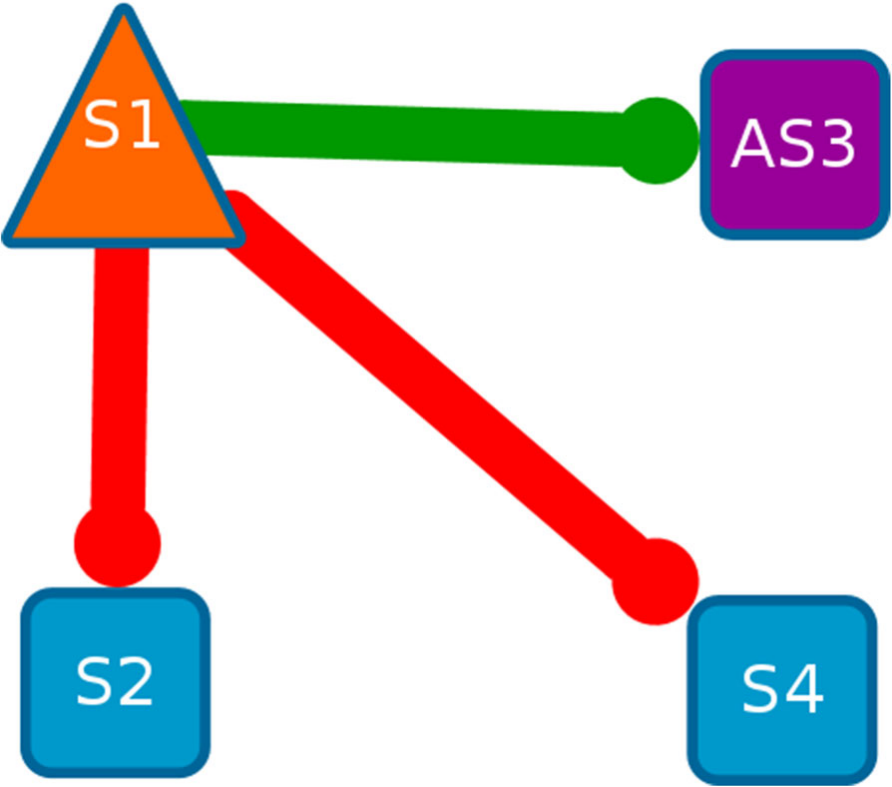
Illustration of a *change motif* in the Extended Core Network. A sense node is represented in blue, an anti-sense node is represented in purple. The orange triangle-shaped node is an *AS-impacted gene*. A red link is only present in the Sense network. A green link is present in the Sense and Anti-Sense network. The link $S1 \multimap S2$ means that the mutual information between *S*1 and *S*2 is maximal for *S*1

We propose a differential network analysis where the extended core network inferred from the sense only data (S) is compared to the extended core network inferred from the sense and anti-sense data (SAS). We focus our analysis on a sense gene that has no sense neighbour in the SAS extended core network. If a sense node has no sense neighbour in the SAS network, it means that the interactions that exist between this gene and others in the S network are not strong enough to be present in the SAS core network. We say that this gene is an *AS-impacted gene*. An AS-impacted gene is observed when the mutual information of this gene with an anti-sense transcript is so strong that, even with the accepting rate, the mutual information between this gene and the other sense transcripts are not significant enough. By representing both S and SAS network in the same graph, we can define a *change motif* as a sub-graph formed by an AS-impacted node, with all its direct neighbours in the S and the SAS networks [32, 43]. We can identify the change motifs directly in the adjacency matrices of both networks. Change motifs allow us to identify local changes on interactions when the anti-sense actors are integrated in the network inference. The information contained in the change motifs shows the importance of taking anti-sense into account because it reveals the interactions between sense and anti-sense transcripts. Figure 3 represents a change motif designed from the AS-impacted node *S*1; the outer links of *S*1 in the S network ($S1 \multimap S2$ and $S1 \multimap S4$) are different than the outer link of *S*1 in the SAS network ($S1 \multimap AS3$).

A change motif surrounding an AS-impacted gene extracts from the data a small set of genes and anti-sense transcripts that deserves a further study. We shall report in the “Results and discussion” section the detailed biological analysis that we have done on these motifs.

#### Steiner trees to compute the rewiring of AS-impacted sub-graphs

Change motifs allow us to have a local view of the impact of the integration of anti-sense actors to the inference method. We now want to have a more global view on this impact. The AS-impacted genes are present all over the S network, but some of them are connected, forming an *AS-impacted sub-graph*. Those AS-impacted sub-graphs represent a part of the network strongly impacted by anti-sense actors. In order to analyse this impact, we study the rearrangement of the AS-impacted gene interactions in the SAS network. In other words, we look for indirect interactions in the SAS network between AS-impacted genes that interacts directly in the S network. This analysis is performed thanks to the Steiner tree problem explained below.

Given an undirected graph *G*=(*V*,*E*) with a set of vertices *V* and a set of edges *E*, and given a subset of vertices *S*⊆*V*, the minimal Steiner tree problem is to find a sub-graph *G*^′^ of *G* such that *S* is contained in *G*^′^, all the vertices are connected by a path, and the number of edges of *G*^′^ is minimal. For a weighted graph, the aim is to minimize the total weight of the edges of *G*^′^. The Steiner tree problem is used to solve several problems, such as extracting information from a large database of molecular interactions [44]. For example, for a given set of proteins, the Steiner tree problem may be applied to the interactome graph with these proteins as terminal nodes to compute a minimal set of relations connecting all these proteins. An experimental study used the Steiner tree problem on a large human protein-protein interaction network [45].

The minimum Steiner tree problem is a NP-complete combinatorial optimization problem [46] and several heuristic methods have been designed to use it with large graphs. In our workflow, we use the shortest paths based approximation [45] that computes the Steiner tree *ST* step by step. The first node added to the Steiner tree *ST* is one of the terminal node. Then, the shortest paths between all the remaining terminal nodes and the nodes of *ST* are computed, and the closest terminal node is connected to the Steiner tree *ST* with the shortest path. Once all the terminal nodes are in the Steiner tree *ST*, the resulting tree is pruned thanks to the minimum spanning tree method.

SteinerNet is a R package that allows to compute the minimum Steiner tree using one exact algorithm, or find a solution using four different approximate algorithms [45]. The shortest paths based approximation is one of the heuristic developed in SteinerNet that can be used on large graphs such as the protein-protein or gene networks. The SteinerNet package was last updated in 2013; then as the dependencies were obsolete, it was removed from the CRAN repository. We updated the package to the R version 3.2.0 and we will be pleased to share this updated package upon request.

In order to analyse the rewiring of AS-impacted genes, we solve the minimum Steiner tree problem with the subset of vertices composed by a AS-impacted sub-graph. AS-impacted sub-graphs have a particularity: all the genes have a direct connection in *S*, but because the genes are AS-impacted, all those connections are not represented in the sense and anti-sense network *SAS*. Thus it is interesting to wonder how these nodes rewired in *SAS*. If we can find a minimal Steiner tree for an AS-impacted sub-graph, the tree show the relationships between the AS-impacted genes of the sub-graph in the sense and anti-sense network. It shows how anti-sense actors intervene in the relationships between connected AS-impacted genes. Several exploitations of the Steiner trees can be done. In one hand, the Steiner tree can be visualized to help focus on interesting interactions. In an other hand, we can perform a functional analysis to identify the main biological functions impacted by anti-sense transcription.

## Results and discussion

### Differential functional analysis

We performed two functional analyses that, for a *p*-value threshold of 0.05, identified GO categories for sense data in one hand and for sense and anti-sense data in an other hand. This differential functional analysis provided 104 revealed-by-AS terms. The list of the revealed-by-AS terms associated with their *p*-value is given in Additional file 1. Among those terms, we can see terms related to cell wall (i.e. “cell wall organization or biogenesis”, *p*-value: 0.004), cold response (i.e. “response to cold”, *p*-value: 0.035) and osmotic response (i.e. “water transport”, *p*-value: 0.001). Those biological functions, related to response to stress and fruit ripening, are consistent with our experimental context since fruits are stored in cold chambers while the ripening continues (the different processes involved in this experiment are more thoroughly explained below). Without the anti-sense data, terms related to such conditions are not in the result of the functional enrichment analysis.

### Differential network analysis

The number of change motifs depends on the accepting rate used in the Extended Core Network method, because this rate determines the neighbourhood size of nodes, and consequently of AS-impacted nodes. We identified 308 change motifs in the 60DAH experiment with an accepting rate of 5%. The number of change motifs is equal to the number of AS-impacted genes, and it represents about 30% of sense actors.

**Fig. 4:**
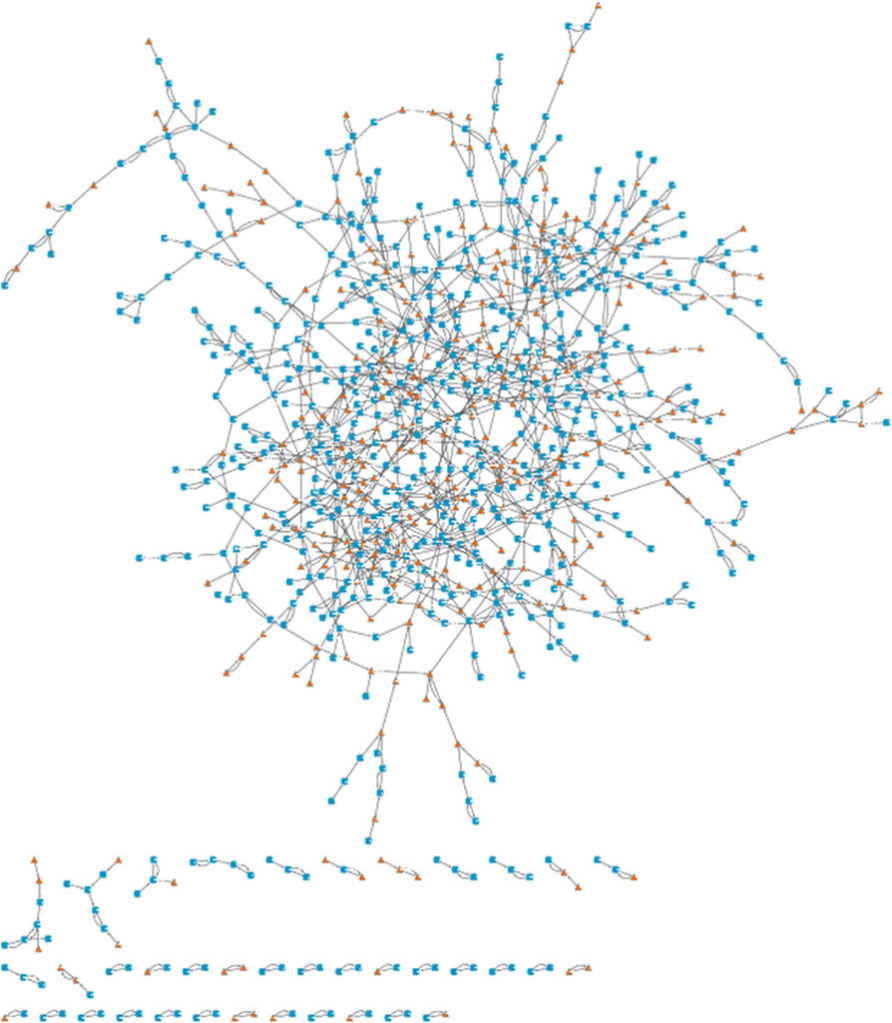
Extended Core Network with a 5% accepting rate for sense-only 60DAH experiment. Orange triangle-shaped nodes represent AS-impacted genes: they are connected to one or several sense nodes in this graph, but in the SAS network, they only have anti-sense neighbors

We performed the differential network analysis on the 60DAH experiment. Figure 4 shows the graphical result of this analysis. We can see the repartition of the AS-impacted genes (orange triangles) and note that they are spread in the network which means that AS transcription impacts is not restricted to a specific set of genes. The graph was drawn using Cytoscape [47] and features of Cytoscape may be used to mine this graph.

### Enrichment analysis of change motifs

We identified 308 change motifs in the 60DAH experiment. Each change motif contains an AS-impacted gene and its direct neighbors in the S network and in the SAS network. For this analysis, each motif is enlarged by also considering the direct neighbours of its anti-sense actors; as the anti-sense actors impact the central gene of the motif, we want to consider the other interactions that they may have in the SAS network. In the following, the expression “change motif” will refer to an enlarged change motif. As a change motif is a gene set produced by our analysis work-flow, it is interesting to know if some biological functions are over-represented by this set. The change motifs are formed on average of 5 genes. Due to this small set size, we perform an enrichment analysis of each change motif using the apple GO slim, which contains 14 categories. Additional file 2 presents the results of this enrichment analysis.

The main post-harvest factors that influence apple softening include temperature, atmosphere, relative humidity, calcium treatment, and ethylene [48]. Artificial prevention of ripening process (and keeping quality) is the main goal of controlled atmosphere storage (low oxygen and high carbon dioxide) and/or cold storage (low temperatures). For cold storage, apples during post-harvest time are stored at 0-4°C. Low temperatures influence the post-harvest biology of apple fruits. Chilling stress is a physiological disorder that limits the storage of chilling sensitive fruits at low, but non-freezing temperatures [49]. Low temperatures disrupt the balance of reactive oxygen species (ROS), leading to its accumulation and oxidative stress [50]. Therefore, in the particular case of the specific transcriptome of apple after two months of cold storage, it is expected to find genes involved in: ripening/cell wall modifications, cold signaling/response to cold stress and response to oxidative stress.

We identified 308 motifs as AS-impacted in the transcriptome of the two months storage set (60DAH). Taking in account the homologies between apple and Arabidopsis genes when available, 72 of them displayed a significant enrichment in the GO slim categories such as “developmental processes”, “response to abiotic or biotic stimulus”, “response to stress” and “transport” (see Additional file 3).

The 291 apple genes involved in the 72 enriched motifs correspond to 209 putative Arabidopsis orthologs. Interestingly, a closer identification of putative biological functions based on the TAIR annotations [34] allowed to relate no less than 127 of them (i.e. 61%) to the above cited processes, in summary: ripening, cold and oxidative stress. They were found in 88% of the motifs (see Additional file 3), indicating that the majority of the genes belonging to change motifs can be directly related to the biological process of fruit post-harvest conservation at low temperature.

Besides, 81 apple genes lack any information on their biological function and are not considered in this analysis. Arabidopsis orthologs of some of them have been cited in deregulated gene lists of various conditions of stress response, which is not inconsistent *per se*, but not highlighted in this study. The consistency of the AS impacted motifs with the apple maturation process under cold temperature can be easily highlighted through the three following examples.

**Fig. 5:**
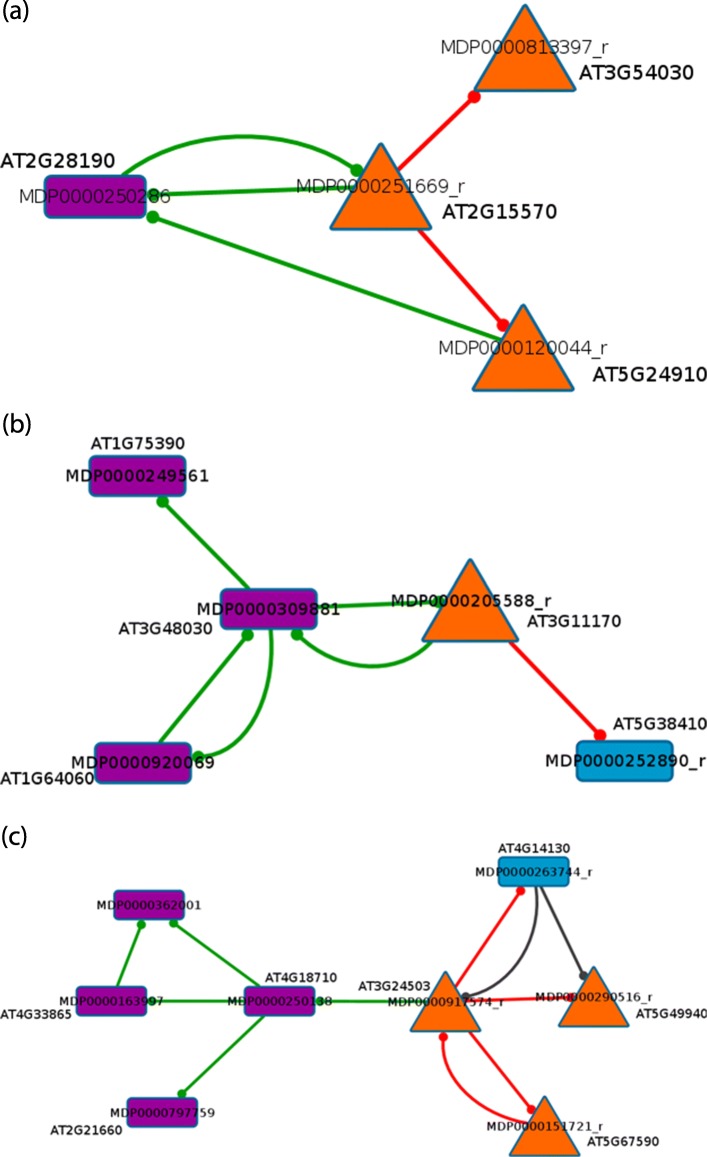
Change motifs from the 60DAH experiment. Orange and blue nodes represent sense nodes, an orange node being an AS-impacted gene. Purple nodes represent anti-sense nodes. A red link is a link only from the sense network. A green link is a link only from the sense and anti-sense network. A gray link is a link from both networks. Each apple gene (MDP) is associated with its best homolog in *Arabidopsis thaliana*. **a** Change motif #1. The AS-impacted gene is MDP 0000251669_r. **b** Change motif #2. The AS-impacted gene is MDP 0000205588_r. **c** Change motif #3. The AS-impacted gene is MDP 0000917574_r

The motif #1 (Fig. 5a) contains 4 genes: MDP0000250286 is encoding for a putative superoxide dismutase responding to cold stress [51], MDP0000120044 is similar to the Cyt P450 monoxygenase CYP714A1 involved in gibberellic acid pathway [52] and MDP0000251669 to a thioredoxin [53], all of them involved in the oxidative stress response. Lastly, MDP0000813397 is encoding a brassinosteroid signaling kinase [54], brassinosteroid being a plant hormone family involved in cold stress response [55]. In this first example, the members of the motif are all directly related to the cold stress response which occurs during the storage process.

Motif #2 (Fig. 5b) contains two genes involved in the response to cold, MDP0000205588, encoding an osmotic fatty acid desaturase [56] and MDP0000249561, the BZIP44 transcription factor also putatively involved in the control of fruit maturation through cell wall loosening [57, 58]; and two genes involved in response to oxidative stress: MDP0000309881, similar to the hypoxia-induced gene domain 1 [59], and MDP0000920069, encoding a respiratory burst oxidase protein F [60] also responsive to ethylene and abscisic acid. Within this motif, only the gene MDP0000252890 encoding a rubisco subunit 3B, could not be directly related to the studied process according to scientific literature.

Out of the eight genes of the Motif #3 (Fig. 5c), five of them are perfectly consistent with the fruit ripening process under cold temperature: MDP0000250138 is an ortholog of BIN2, a member of the ATSK (shaggy-like kinase) family which acts in the cross-talk between auxin and brassinosteroid signaling pathways [61], MDP0000797759, a glycine-rich RNA binding protein which increases stress tolerance under conditions of low temperature [62], MDP0000151721 is an ortholog of Fro1 involved in cold acclimation and osmotic stress response [63], MDP0000263744, a xyloglucan endotransglycosylase involved in cell wall modification and cold acclimation [64], and finally MDP0000917574, an aldehyde dehydrogenase 1A which is a critical gene of the phenylpropanoid pathway involved in the production of antioxidant components and the response to biotic and abiotic stresses [65].

In summary, these results show that the motifs obtained by taking in account the AS transcriptome in the network inference highlights new actors. In this case we clearly show that biological functions of these new actors can be related to the studied biological question. Therefore the consideration of these new actors, either sense transcripts, or anti-sense transcripts supposed to act as regulators of the corresponding coding genes, sheds a new light of the putative regulation networks underlying the studied processes.

Moreover, a synthetic view of the whole set of AS-impacted motifs can provide new avenues of work to point pathways or genes whose importance could have been underestimated without this input. In this case it is noteworthy that at least 11 occurrences of genes related to the brassinoids pathway appear in the set of 72 motifs. Brassinoids have been reported to be involved in a range of developmental processes, such as stem and root growth, floral initiation, and the development of flowers and fruits [55, 66]. Studies also revealed that brassinoids can confer resistance of plants to various abiotic and biotic stresses, including cold stress [67, 68]. Li et al. [69] even reported that brassinolide mediates tolerance of plants to abiotic stress in general and cold stress in particular. Brassinoids have also been reported as involved in grape berry ripening [70] and early fruit development in cucumber [71], but their implication has not been reported yet in apple development and maturation. The present study has shown that several genes involved in the brassinoid pathway might play a role in the apple maturation and conservation at low temperature.

### AS-impacted sub-graphs

**Fig. 6:**
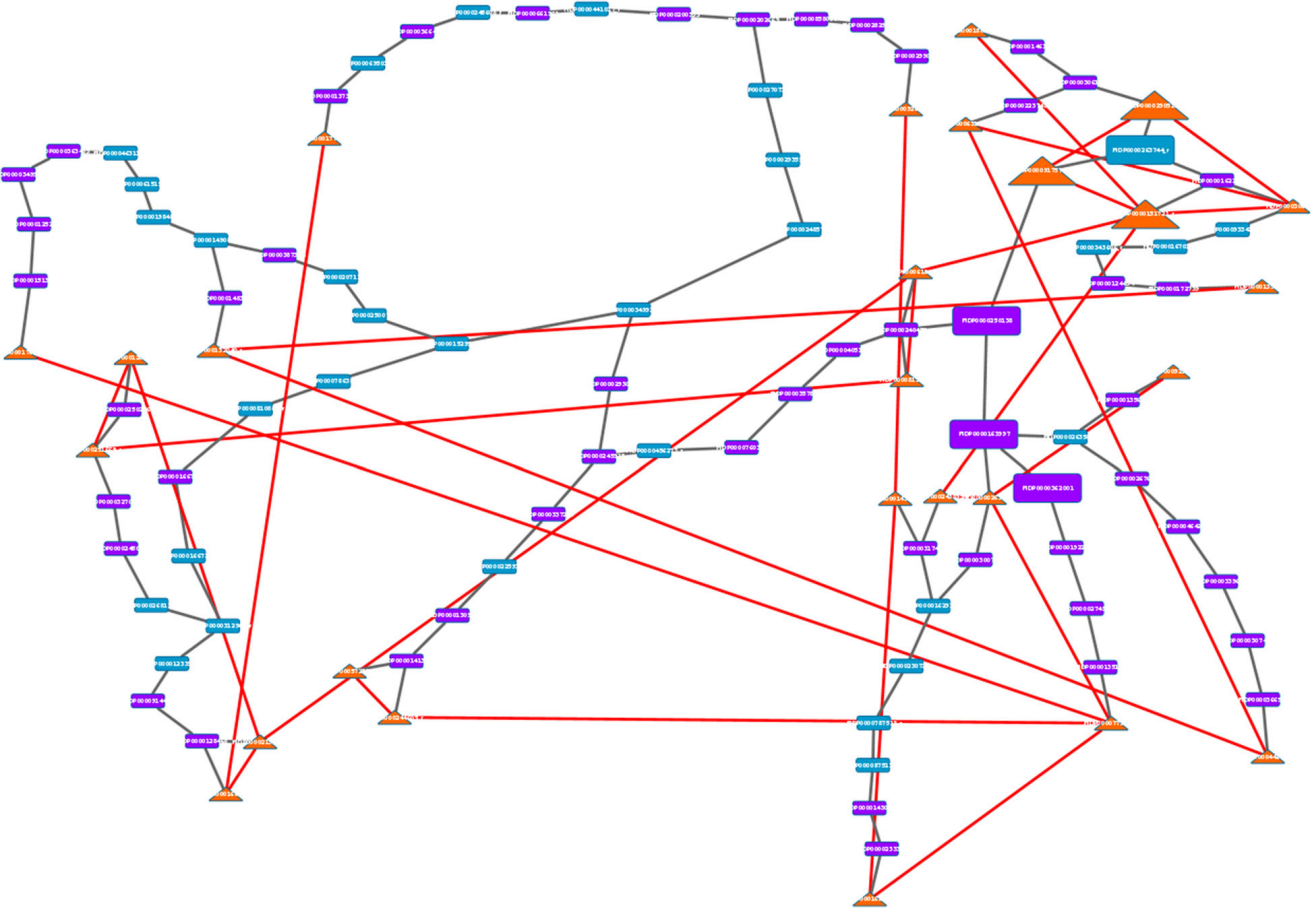
Steiner tree of an AS-impacted sub-graph from the 60DAH experiment. Orange nodes are AS-impacted genes, blue nodes are Steiner sense nodes, purple nodes are Steiner anti-sense nodes. Gray links are connections from the SAS network, red links are connections from the S network. Bigger nodes corresponds to the nodes from the change motif #3

In the 60DAH experiment we identify 29 AS-impacted sub-graphs, when considering only graphs with at least three connected AS-impacted nodes. For instance, Fig. 6 shows a Steiner tree containing the AS-impacted gene of the change motif #3. This Steiner tree connect all the AS-impacted genes (drawn in orange triangle-shaped nodes) thanks to sense (blue) and anti-sense (purple) nodes from the SAS network. Red links are interactions from the S network impacted by anti-sense transcription; they do not appear in the SAS network (nor in the Steiner tree). With this visualization, we can see that the core of the network has been highly modified by the integration of anti-sense actors, however AS-impacted genes that were direct neighbors in the S network are still connected but in an indirect manner. The Steiner tree from Fig. 6 is composed by 26 AS-impacted genes and 82 Steiner nodes. We perform a functional enrichment of AS-impacted genes in one hand and the whole genes of the Steiner tree in an other hand using the GO slim. The 26 AS-impacted genes are tagged with two GO slim categories: “other metabolic processes” (*p*-value: 0.030) and “transcription, DNA-dependent” (*p*-value: 0.023). The functional enrichment of the 108 genes of the Steiner tree are enriched by the “response to abiotic or biotic stimulus” GO slim category with a *p*-value of 0.048, which is consistent with our experimental context.

## Conclusion

The original proposition of our work is to analyse the impact of anti-sense transcription on a large scale. To achieve this goal, sense and anti-sense transcripts are treated in the same way in our gene network inference method. By considering the recent ideas of differential network analysis, our main proposal is to compare network inferred from different sets of data: the sense data in one hand, and both the sense and anti-sense data in an other hand. To compare the inferred networks, we first propose the Extended Core Network inference method. Secondly we define the differential network analysis that we performed with the Extended Core Network method. The comparison of two networks inferred on different sets of actors shows that the integration of the anti-sense data clearly modifies the network topology. We define the AS-impacted genes and the change motifs, that identify those changes in the network topology. The biological analysis of change motifs on apple data highlights interesting actors and emphasizes the interest to take anti-sense data into account in transcriptomic analysis. The differential network analysis identifies subsets of new actors in the context of maturation and conservation of the fruit that will be explored in further studies. We also propose to study the AS-impacted sub-graphs to provide a more global view of the impact of anti-sense transcription on biological functions.

## Additional files

Additional file 1 List of the GO categories revealed by anti-sense data. List of the 104 revealed-by-AS terms. Column A: Identifier of the GO category. Column B: significant *p*-value. Column C: significant corrected *p*-value below 0.05, the correction used is the Benjamini & Hochberg False Discovery Rate (FDR) correction (Revealed-by-AS terms are ordered according to this criteria). Column D: Number of apple genes from the sample tagged with the GO category. Column E: Number of apple genes from the genome tagged with the GO category. Column F: Number of apple genes in the sample. Column G: Number of apple genes in the genome. Column H: Name of the GO category. Column I: Apple genes from the sample tagged with the GO category. Column J: Arabidopsis orthologs of the apple genes from the sample tagged with the GO category; here an Arabidopsis ortholog with an anti-sense apple gene is identified by the “_AS” prefix. (ODS 57 kb)

Additional file 2 Change motifs enrichment. Results of the enrichment analysis of the 308 change motifs from the 60DAH experiment. Columns A to D: apple genes, nodes of the change motif. Column E: GO slim category significantly over-represented by the genes present in the change motif (“No enrichment” if there is no significantly over-represented category). Column F: significant *p*-value below 0.05 (-1 if no enrichment). Column G: Apple genes from the sample tagged with the GO slim category. Column H: Number of apple genes in the genome. Column I: Number of apple genes in the sample. Column J: Number of apple genes from the genome tagged with the GO slim category. Column K: Number of apple genes from the sample tagged with the GO slim category. Columns L to O: Arabidopsis orthologs of the apple genes of the change motif. Column P: Arabidopsis orthologs of the apple genes from the sample tagged with the GO category. (ODS 34 kb)

Additional file 3 Functional annotation of change motifs significantly enriched according GO slim classification. For each enriched change motif from Additional file 2, we associate functional keywords from the TAIR annotation. Columns A to D: apple genes, nodes of the change motif. Column E: GO slim category significantly over-represented by the genes present in the change motif. Column F: significant *p*-value below 0.05 (change motifs are ordered according to this criteria). Column G: Arabidopsis orthologs tagged by the GO slim term associated with the change motif. Columns H to N: Functional keywords from Arabidopsis orthologs (TAIR annotations), keywords consistent with the biological context (fruit ripening in abiotic stress condition) are highlighted in orange. In columns H to N, we use the following abbreviations: ABA: abscissic acid; SA: salicylic acid; JA: jasmonate acid; ROS: reactive oxygen species. The three change motifs described in the manuscript (Fig. 5) are highlighted in blue. (ODS 23 kb)

Additional file 4 H experiment — http://www.info.univ-angers.fr/%7elegeay/AF4%5fHarvest.zip. Normalized data (without background suppression) of the H (Harvest) experiment. The file contains the normalized expression data of the 126,022 apple genes (rows) of the 22 samples (columns). The normalization method used is the quantile normalization. (TXT 1 kb)

Additional file 5 60DAH experiment — http://www.info.univ-angers.fr/%7elegeay/AF5%5f60DAH.zip. Normalized data (without background suppression) of the 60DAH (60 Days After Harvest) experiment. The file contains the normalized expression data of the 126,022 apple genes (rows) of the 22 samples (columns). The normalization method used is the quantile normalization. (TXT 1 kb)

## Abbreviations

AS: Anti-sense
C3NET: Conservative causal core network
ECN: Extended core network
GO: Gene ontology
RNA: Ribonucleic acid
S: Sense
SAS: Sense and anti-sense
